# Editorial: A gendered approach for accelerating prevention and control of NCDs

**DOI:** 10.3389/fpubh.2024.1484592

**Published:** 2024-09-19

**Authors:** Aswathy Sreedevi, P. S. Rakesh, Sairu Philip

**Affiliations:** ^1^Amrita Institute of Medical Sciences and Research Centre, Amrita Vishwa Vidyapeetham University, Kochi, India; ^2^Harvard T.H. Chan School of Public Health, Harvard University, Boston, MA, United States; ^3^The Union, New Delhi, India; ^4^Government Medical College, Kottayam, Kerala, India

**Keywords:** gender, non-communicable diseases, risk factors, prevention, control

Gender is an important social determinant that shapes health behaviors, exposures, vulnerabilities, and influences health systems' responses (1). The first step in managing this is to acknowledge that women and men are not homogenous groups, and that their health opportunities and risks vary according to social, economic, environmental, and cultural influences (2).

Planning gender-responsive health promotion or preventive campaigns and interventions requires an understanding of the role of sex and gender in the burden of Non-Communicable Diseases (NCDs) and their behavioral risk factors. Current Research Topic on “*A gendered approach for accelerating prevention and control of NCDs*” has 15 articles which primarily focuses on the sex and gender differences with regard to morbidity and mortality of NCDs, its behavioral risk factors and access to care. This Research Topic is demographically and geographically diverse with studies among varying age groups from pre-school children to older citizens and from various countries across six continents.

The analysis of gender disaggregated data is the first step in mainstreaming gender in any health issue. Collecting and analyzing data disaggregated by sex is essential to identify differentials between women and men, whether related to sex or gender, in terms of disease magnitude and severity as well as its outcomes. Current Research Topic has four studies describing the gender disaggregated burden of NCDs. Study by Yue et al. revealed gender disparity and temporal trends of liver cancer in China from 1990 to 2019. Liver cancer incidence was three times higher in males than females and most of the cases occurred in males aged 50–54 years and females from 65 to 69 years. Felsinger et al. highlighted gender differences in the lung cancer epidemiology in Austria with the mean age of death of male lung cancer patients showing a consistent increase from 1992 to 2921, whereas women showed no significant change from 1992 to 2021 indicating that smoking behavior among adolescents girls should be tackled. Guzzinati et al. have done a retrospective population-based cohort study with 9,726 cutaneous malignant melanoma survivors and differentially assessed the risk of synchronous and metachronous cancers stratified by sex. Both sexes demonstrated an excess risk for synchronous kidney/urinary tract malignancies. Women had an increased risk of synchronous breast cancer and males had an increased risk of metachronous thyroid and prostate malignancies. Migrants from West Africa, in Spain also showed marked gender-wise differences in morbidity between females and males for various NCDs and metabolic risk factors (MacKinnon et al.). Being female increased the risk of NCD and its risk factors, in addition to others such as living in Spain for >5 years, and being aged >50 years.

Using an economic model to quantify the impact of tobacco-related illnesses, Alcaraz et al. explored the differences in tobacco-attributable disease and economic burden between men and women in Argentina, Brazil, Chile, Colombia, Costa Rica, Ecuador, Mexico, and Peru. Risk factors for NCDs are strongly influenced by gender (1). There is a need to address the impact of gender norms and roles on the differential exposure to risk factors between men and women. Exploring magnitude and trends in exposure to risk factors will provide valuable insights into the prevention and control of non-communicable diseases. The current Research Topic has six articles in this regard. Mayer et al. analyzed data of 1,891 pre-school children from 224 early childhood education and care centers from Germany and provided gender disaggregated data and highlighted health inequalities among the children. A social gradient was observed with a higher BMI among children from lower socioeconomic groups families. Luo et al. looked at the gender differences in family meal frequency and the relationship between meal frequency and other health measures, among 159,904 California middle and high school students. Less than half (44.7%) students reported a high frequency of family meals and boys were more likely to have more frequent family meals compared to girls and those who identified their gender in another way. Being a girl was associated with a lower frequency of family meals and these differences started in middle school and persisted regardless of good family relationship. The findings were worse for those who did not identify as male or female.

Baj-Korpak et al. looked at the physical activity levels and kinesiophobia in medical students from Poland and Belarus during the COVID-19 pandemic. Male medical students in Poland had higher physical activity in-spite of restrictive conditions during COVID-19, whereas in Belarus with no restrictive conditions in force girls were more kinesiophobic. Yu Y. et al. explored the potential risk factors and gender differences for physical activity-related injuries (PARI) amongst 6,032 students in rehabilitation from 90 universities in China. Males had a higher cumulative frequency of injury and high levels of physical activity were associated with a greater risk of PARI. In addition high antisocial risk scores were also associated with elevated risk of PARI. In addition among girls, region wise differences were observed with girls from west China having a lower risk of PARI compared to East China. The study provides a basis for developing future injury prevention mechanisms with attention to differences between genders. In Kazhakhystan, Baspakova et al. provided an overview of the prevalence and associated behavioral risk factors for NCDs. Men showed higher rates of smoking and alcohol use, while women exhibited a greater prevalence of physical inactivity and obesity.

Health-seeking behavior in a gender/sex disaggregated manner could also give meaningful insights into the role of gender. Globally, women face significant barriers to access timely, adequate, or affordable health screening and care (3).

Yu H. et al. explored the experiences of older women with acute myocardial infarction (AMI), focusing on their perception, challenges, and coping strategies at the onset of chest pain in China. The study provides recommendations to reduce older women's delay in seeking care and highlighted the need for addressing gender related disparities among MI patients in accessing care. Targeted interventions to increase breast cancer screening attendance among women above 60 years has been suggested by Pedrós Barnils and Schüz who performed an intersectional analysis of inequalities in self-reported breast cancer screening in Spain. This often results in poor health outcomes and high rates of deaths among women in low-resource settings (3). Montesó-Curto et al. have qualitatively explored men's emotional experiences with fibromyalgia in clinics in Spain and the U.S., providing valuable insights into this area. The study highlighted the need for incorporating the emotional management into all treatment protocols for fibromyalgia, especially for men given the gender stigma. All the three studies will add to the evidence base and rationale to strengthen health systems responses to control NCDs.

Women face many gender-based challenges in relation to chronic diseases, especially in low- and middle-income countries (4). Nair et al. assessed the quality of life of postmenopausal women in India and described the risk of osteoporosis among them. The study underscored the complex interplay of demographic factors, menopausal experiences, and their impact on the participants' quality of life.

Violence against women is now recognized as a public health problem of epidemic proportions. A recent review explored link between violence and NCDs from a chronic stress framework and have found that survivors of repeated violence present an increased risk of NCDs (5). Debel et al. evaluated the prevalence and factors associated with gender based violence (GBV) among 6,085 female sex workers (FSW) in Ethiopia. The findings will have significant implications for program planning on prevention and response to mitigate the occurrence and impact of GBV among FSWs. The present Research Topic of articles primarily includes non-communicable diseases and its risk factors. Other than the conventional Non-communicable diseases (NCDs) such as cardiovascular diseases, cancers, mental health disorders, some rare chronic diseases such as fibromyalgia, violence have also been included. Violence is a non-communicable disease in the context of medicine and public health (6). The common risk factors for all non-communicable disease would appear to make the mitigation easier. However, several cross cutting factors such as gender, socioeconomic status make the mitigation difficult at the best of times. Understanding the interaction and complex contexts can be a first step toward mitigation. Several articles in this Research Topic give a varied view of the NCD's its risk factors among various age groups.

To summarize, the Research Topic reiterates that NCDs does not affect genders equally, as there are variations in prevalence, risk factors, and access to healthcare services among different gender groups. It is essential to comprehend these differences in order to formulate targeted interventions and policies that cater to the specific requirements of each gender. Gender-responsive actions are needed to prevent, detect, manage and control NCDs and is crucial to ensure an equitable response in line with Universal Health Coverage and the Sustainable Development Goals.
